# Comparability of The Netherlands Physical Activity Questionnaire with Accelerometer-Measured Physical Activity in Samoan Children: A Retrospective Analysis of *Ola Tuputupua’e* Data

**DOI:** 10.3390/ijerph18168438

**Published:** 2021-08-10

**Authors:** Clara R. Warmath, Courtney C. Choy, Elizabeth A. Frame, Lauren B. Sherar, Rachel L. Duckham, Christina Soti-Ulberg, Take Naseri, Muagututia S. Reupena, Nicola L. Hawley

**Affiliations:** 1 Department of Epidemiology, School of Public Health, Brown University, 121 South Main Street, Providence, RI 02906, USA; clara_warmath@brown.edu (C.R.W.); courtney_choy@brown.edu (C.C.C.); taken@health.gov.ws (T.N.); 2 Department of Epidemiology, University of Michigan School of Public Health, 1415 Washington Heights, Ann Arbor, MI 48109, USA; lizframe14@gmail.com; 3 Center for Global Health and Human Development, School of Sport and Exercise Sciences, Loughborough University, Loughborough LE11 3TU, UK; l.b.sherar@lboro.ac.uk; 4 Institute for Physical Activity and Nutrition (IPAN), Deakin University, 221 Burwood Highway, Burwood, VIC 3125, Australia; duckhamrl@gmail.com; 5 Australian Institute for Musculoskeletal Science (AIMSS), The University of Melbourne and Western Health, 176 Furlong Road, St. Albans, VIC 3021, Australia; 6 Ministry of Health, Ififi Street, Motootua, Apia, Samoa; christinau@health.gov.ws; 7 Lutia i Puava ae Mapu i Fagalele, Apia, Samoa; smuagututia51@gmail.com; 8 Department of Chronic Disease Epidemiology, Yale School of Public Health, 60 College Street, New Haven, CT 06520, USA

**Keywords:** physical activity, Samoa, infancy and childhood, accelerometry, Netherlands Physical Activity Questionnaire

## Abstract

Accurate measurement of physical activity is critical to understand its role in cardiometabolic health and obesity development in children and to monitor trends in behavior and evaluate interventions. An ongoing mixed-longitudinal study of child growth and development in Samoa is collecting physical activity data with both accelerometers and the Netherlands Physical Activity Questionnaire (NPAQ). The aims of our analyses were to (1) describe the response frequency and correlations of individual questions in the NPAQ, (2) develop modified NPAQ scores with selected questions and (3) examine the concordance of modified NPAQ scores with accelerometer outcomes among children aged 2–4 years. We developed two modified NPAQ scores with combinations of questions and assessed concordance of the modified scores with accelerometer data using estimated marginal means adjusted for monitor wear time. Although the evenly distributed tertiles of the modified 15-point NPAQ score showed promising trends of increasing minutes of accelerometer-assessed high-intensity physical activity with increasing tertile, the estimated marginal means were imprecise with high variance, demonstrating that NPAQ score could not accurately assess physical activity levels of preschool-aged children in Samoa. Considering that questionnaires are often considered more cost-effective tools for physical activity measurement than accelerometry, further research is necessary to develop a culturally and age-appropriate physical activity questionnaire in this population.

## 1. Introduction

There is a growing burden of childhood overweight and obesity in low- and middle-income countries, with Pacific Island nations experiencing one of the highest increases in prevalence in the world. The 2020 edition of UNICEF/World Health Organization (WHO)/World Bank Group’s Joint Child Malnutrition Estimates reports that between 2000 and 2019, the prevalence of overweight among children under five in Oceania rose from 4.7% to 9.4% [1].

As well as having many long-term cardiovascular benefits [2,3], physical activity plays a crucial role in the prevention of obesity and noncommunicable diseases. In a multiethnic study of children aged 3–15 in the US, for every additional day of the past seven days that children were physically active for ≥60 min, there were 7% lower odds of overweight or obesity (based on body mass index (BMI) ≥85th percentile using the US Center of Disease Control’s sex-specific 2000 BMI-for-age growth charts) [4]. Despite the potential benefits of physical activity to prevent obesity in this high-risk population, little is currently known about the baseline physical activity levels of preschool-aged children in the Pacific region, nor are there recommended tools for its measurement. Further, the WHO recommends that children aged 2–4 years “spend at least 180 min in a variety of types of physical activities at any intensity, of which at least 60 min is moderate- to vigorous-intensity physical activity [by age 3], spread throughout the day; more is better”; however, the evidence base for the guidelines included few studies from the Pacific region [5]. Additional data collection is needed to evaluate the guidelines’ utility for region-specific health promotion.

Accelerometry is promoted for its “objective” measurement of physical activity [6] but can be costly and impractical to implement for some large population studies. Consequently, physical activity is often estimated using questionnaires; however, issues with acute recall and reporting and social desirability bias are of significant concern [7]. These methodological challenges are even greater for young children due to the reliance on proxy reporting in this age group. There is a general lack of validated physical activity questionnaires for use in young children [8]. In addition, there has been little testing of questionnaire performance/validity for children in low- and middle-income countries. The reliability and consistency of physical activity questionnaires may be highly context dependent due to cultural and socioeconomic differences between population settings. Questions to estimate physical activity levels in young children often incorporate descriptions of behaviors associated with high or low physical activity levels in high-income countries where questionnaires are initially developed. For example, many questionnaires inquire about participation in sports and group play given high enrollment in developmental programs and organized sports at a young age in many high-income countries [9]. In addition, reading and screen time are often used as proxies for estimating sedentary time in children. In low- and middle-income countries, these indicators may not adequately capture childhood activity given the limited access to computers with internet access and age-appropriate reading material [10]. Moreover, varying cultural perceptions of physical activity may also impact how mothers report the physical activity of their children. 

In 2015, we chose to use the Netherlands Physical Activity Questionnaire (NPAQ) in an ongoing mixed-longitudinal study of child growth and development in Samoa [10] because, at the time, it was one of the only available questionnaires for the target age range of interest (two to four years of age). The NPAQ had previously been used in other low- and middle-income country settings such as Brazil [11,12] and is less focused on structured play and organized sports than other questionnaires, which we considered more appropriate for the social and cultural context of Samoa.

The aims of this retrospective analysis were to (1) describe the response frequency and correlations of individual questions in the NPAQ (2) develop modified NPAQ scores with selected questions and (3) examine the concordance of modified NPAQ scores with accelerometer data among children aged 2–4 years.

## 2. Materials and Methods

### 2.1. Study Design and Participants

The *Ola Tuputupua’e* (“Growing Up”) study is a mixed-longitudinal study examining determinants of child growth, health and development in Samoa. Recruitment of the *Ola Tuputupua’e* cohort took place between June and August 2015 on the Samoan island of Upolu and has been previously described [13]. Briefly, a convenience sample of 319 mother–child pairs was recruited from 10 villages on the island of Upolu, the most populated of the Samoan Islands, where >75% of the population are residents [14]. With the intention of understanding the health impacts of differential exposure to the nutrition transition, the distribution of the sample was intended to equally represent each of the three Upolu census regions (4 villages from the Apia Urban Area with the highest exposure, 3 villages from Northwest Upolu and 3 villages from the rest of Upolu with the least exposure). 

At the time of recruitment, eligible mothers were over 18 years of age, not pregnant and reported no severe physical or cognitive impairments. Eligible children were between 2.00 and 4.99 years of age, of Samoan descent (based on the maternal report of the child having four Samoan grandparents) and had no maternal-reported physical or cognitive impairments.

All study procedures were reviewed and approved by the Yale and Brown University Institutional Review Boards and the Health Research Committee of the Samoan Ministry of Health (IRB Number 2000020519 and IAA Number 18-41 959, April 2015). All participants provided written informed parental consent.

### 2.2. Questionnaire-Based Data Collection

Questionnaires were administered to all mothers in Samoan by trained Samoan interviewers to collect information on various child, maternal and household-level characteristics. Mothers were asked to report whether their child was currently attending school, the years of education that they received and ownership of consumer durable items (such as a TV and refrigerator) in the household to calculate a household asset score, with higher scores indicating greater socioeconomic resource levels [13].

Mothers were asked to respond to the NPAQ to assess the physical activity levels of their children (Appendix A) [15]. Each of the seven NPAQ items had two statements situated on the opposite ends of a spectrum (e.g., “The child prefers to play inside” and “The child prefers to play outside”). Mothers scored their child on a Likert scale from 1 to 5 based on how strongly their child’s behavior aligned with either statement, with 1 typically corresponding with the less active option and 5 typically corresponding to the more active option. The scales for Questions 2 and 6 were reverse coded for scoring. A total score (out of 35) was calculated by summing the scores for each question, with the highest scores theoretically indicating the highest physical activity level.

### 2.3. Accelerometer Data Collection and Processing

Accelerometer-based estimates of physical activity were collected in a randomly selected subset of the sample (*n* = 61) using triaxial Actigraph wGT3X-BT devices set to collect data at a resolution of 30 Hz (ActiGraph Corporation, Pensacola, FL, USA) [16,17]. Each child was asked to wear an accelerometer continuously (i.e., for 24 h, not removing for sleep) for six days before returning it to the research staff. The accelerometers were attached to the children’s nondominant wrists with waterproof hospital bands to prevent them from being removed during the course of the study. Placement of accelerometers on the wrist was considered more feasible for the Samoan setting and likely to enhance compliance compared with the common waist placement given the clothing style, climate and frequent engagement in water-based activities. ActiLife software (version 6.13.1, ActiGraph Corporation, Pensacola, FL, USA) was used to reduce the data into 5 s epochs, and KineSoft software (version 3.3.80, Eslinger Consulting, Clifton Royal, New Brunswick, Canada) was used to process the data. Nonwear was defined as 60 min or more of consecutive zero counts, allowing for 2 min nonzero interruptions. A valid day was defined as greater than 420 min of valid wear between 7:00 a.m., the average wake time reported by mothers, and 9:00 p.m., the average going to sleep time reported by mothers. Our analyses excluded the first day of wear due to a lack of consistency in the deployment times to control for any initial changes in behavior due to the device (i.e., reactivity). The average number of valid days was 5.4 (range: 1–7). There was no association between the number of valid days and physical activity parameters; therefore, to maximize sample size, we included all participants that had at least one day of valid data in our analyses. Intensity cut points defined by Johansson et al. [18] were used to classify count data into outcome variables: sedentary (≤221 counts/5 s), low-intensity (≥221 and <729 counts/5 s) and high-intensity (≥730 counts/5 s) minutes per day. Raw accelerometer data were expressed as average counts per minute (CPM; i.e., total counts per valid day divided by monitor wear time per valid day).

### 2.4. Statistical Analyses

We first described the characteristics of the total sample recruited in 2015 and the subsample of children who wore accelerometers to assess how representative the children who wore accelerometers were of the whole cohort. To assess for differences between the total and subsample, we used *t*-tests for continuous variables with normal distributions, Wilcoxon–Mann–Whitney tests for continuous variables with non-normal distributions and Chi-Square or Fisher’s Exact tests for categorical variables.

Given that the NPAQ physical activity scores did not differ between boys and girls, we chose not to stratify our analysis by sex to maximize sample size. We then examined the response frequency distribution for the seven NPAQ questions in order to determine whether there was sufficient variation in responses for the questions to have a discriminative ability. Because score distributions were not normal, we assessed the relationships between each NPAQ question using Spearman correlations, and 95% confidence intervals were calculated for the correlation coefficient using the RVAideMemoire package [19].

Considering the different scales of physical activity measurement, with accelerometer data on a continuous scale and NPAQ scores on an ordinal scale, we used generalized linear regression analyses to estimate marginal means of accelerometer-measured physical activity and examine trends with maternal-reported NPAQ scores for individual questions. For concordance, we expected higher accelerometer-measured vigorous activity per minute with a higher NPAQ score or higher accelerometer-measured sedentary time per minute with a lower NPAQ score.

Based on both the response frequencies of the NPAQ question and their observed concordance with accelerometer activity data, we then developed alternative NPAQ scores with different combinations of the NPAQ questions. When deciding which questions to include in our modified scores, we looked for the strongest correlations between the NPAQ questions, evenly distributed response frequencies and trends of increasing or decreasing estimated marginal means of accelerometer data according to NPAQ scores (see Section 3). To examine the scale reliability and internal consistency of each modified NPAQ score, we estimated Cronbach’s alpha and compared it to the full scale. These values were calculated in R using the ltm package [20,21]. We divided our sample into quantiles based on their modified scores given the non-normal distribution of the data and to allow for ease of interpretation.

Finally, we assessed the concordance of our newly generated 15- and 30-point scores with accelerometer-measured CPM, vigorous-activity minutes per day and sedentary minutes per day using estimated marginal means adjusted for average daily wear time. We performed this analysis with several different cut points: evenly distributed tertiles and quartiles, as well as tertiles and quartiles determined by Likert score groupings (e.g., Likert scores groupings of 1–2, 3–4 and 5 corresponded to cut points of 1–6, 7–12 and 13–15 for the 15-point score). It was not possible to generate evenly distributed quartiles for the 15-point modified score due to the score distribution (42.6% of the sample scored 15 of 15 points). A two-sided alpha of 0.05 was specified. Estimated marginal means and standard errors were presented for generalized linear models. All analyses were conducted in R version 3.6.1 [21].

## 3. Results

### 3.1. Sample Characteristics

The average age of children who received accelerometers (3.45 years old) was similar to that of the full sample (3.31 years old, Table 1). In addition, the children in the randomly selected accelerometer subsample and the full sample were similar in terms of the distributions of child sex, maternal characteristics, household assets and census region of residence.

### 3.2. Correlations between Individual Questions of NPAQ and Their Concordance with Accelerometer-Measured Physical Activity

For each of the seven questions in the NPAQ, we observed variations in response frequencies, Spearman correlations between questions and concordance with accelerometer data (Table 2). The response frequencies largely favored the extremes of the Likert scale. For each question except for Question 5 (likes/dislikes reading), over half of the sample chose the response indicating the highest level of physical activity. This finding was most obvious for Question 1 (prefers to play with other children/alone), where 94% of respondents answered that their child preferred to play with other children versus playing alone. Correlations between questions varied from weak to moderate, ranging from −0.09 to 0.34 (Table 3). Notably, Question 5 (likes/dislikes reading) was weakly correlated with all the other questions. When we explored the concordance of each question with average accelerometer CPM, average minutes of high-intensity activity per day and the average minutes of sedentary time per day (Table 4), there was a lack of distinct trends. 

### 3.3. Development of Modified NPAQ Scores

We calculated both 30-point and 15-point modified NPAQ scores using the questions we deemed to have the greatest degree of agreement with accelerometer-measured physical activity. The 30-point score included all questions except Question 1. Considering that 94% of the cohort answered 5 for Question 1 (prefers to play with other children/alone), this question did not differentiate between activity levels and was excluded from both modified scores (Table 2). The 15-point score included Questions 2 (prefers vigorous/quiet games), 4 (extroversion/introversion) and 6 (likes to play outside/inside) because of their relatively stronger correlations with each other when compared to correlations between other questions (Table 3, Spearman’s rho > 0.2), as well as their alignment with the accelerometer data (Table 4). Questions 3 (likes/dislikes playing sports) and 5 (likes/dislikes reading) were excluded from the 15-point score due to concerns that their respective focuses on organized sport and reading were not applicable to the age group being studied nor culturally relevant in this population. Question 7 (less/more physically active compared to other children) was excluded from the 15-point score because the observed trends between the NPAQ responses and the accelerometer data were the reverse of what was expected.

When we examined Cronbach’s alpha for each modified NPAQ score, internal consistency increased from the full NPAQ score (0.479) to the 15-point score (0.694). Conversely, there was a slight decrease in internal consistency from the full NPAQ to the 30-point score (0.438).

### 3.4. Concordance of Accelerometry and Modified Scores

With increasing evenly distributed tertiles, the 15-point score exhibited larger increases in estimated marginal means for average daily CPM and average high-intensity minutes per day than the 30-point score (Table 5). Taking a closer look at the difference in means between the first and third evenly distributed tertiles, using the 15-point score resulted in an increase of 219 CPM per day, an increase of 10 min of high-intensity activity per day and a decrease of 9 min of sedentary time per day, while the 30-point score resulted in an increase in 73 CPM per day, an increase of 4 min of high-intensity activity per day and a decrease of 3 min of sedentary time per day. With high variance indicated by the standard errors, neither modified NPAQ score category was adequately indicative of children’s accelerometer-measured physical activity.

## 4. Discussion

The full NPAQ did not exhibit acceptable levels of concordance with accelerometry to evaluate physical activity levels in Samoan children aged two to four years old. Although the evenly distributed tertiles of the modified 15-point NPAQ score showed promising trends of increasing minutes of accelerometer-assessed high-intensity physical activity with increasing tertile, the estimated marginal means were imprecise, demonstrating that the modified NPAQ scores also could not accurately assess the physical activity level of preschool-aged children in Samoa. For these reasons, despite the higher cost, we believe that accelerometry should be used to quantify the intensity and duration of physical activity in this population until more accurate questionnaire-based measures can be developed.

Our findings are consistent with the few other studies that have aimed to validate the NPAQ in different populations [11,12,22,23]. An analysis of children four to seven years old in Iowa, for example, found similarly poor validity when examining Spearman correlation coefficients between NPAQ scores and accelerometer-measured vigorous-activity minutes per day [22]. Interestingly, the strength of the correlations of individual questions with accelerometer data differed significantly from our findings; Janz et al. found Questions 3 (likes/dislikes sports) and 7 (less/more physically active compared to other children) to be the most correlated with accelerometer data in their population, while we determined that Questions 2 (prefers vigorous/quiet games), 4 (extroversion/introversion) and 6 (likes to play outside/inside) were the most aligned with accelerometer data in our population (although we acknowledge that the correlations were not strong). These differences underscore the necessity of considering context when identifying questions that may be most discriminative for any given population.

There are important methodological considerations when administering the NPAQ. Because of the inability of children to complete the questionnaire themselves, the NPAQ depends on mothers to accurately describe their child’s physical activity preferences. Maternal reporting, as well as other forms of proxy reporting, can result in overestimations of physical activity in preschool-aged children due to assumptions that preschool children are highly active [24]. The physical activity of preschool-aged children may be more integrated into daily life rather than relegated to leisure and recreational time, which may make them harder for mothers to identify and recall for reporting. Specific to the social and cultural context of Samoa, families often engage in shared child rearing, and much of young children’s physical activity may occur outside of the home environment [25]. This cultural practice poses a challenge for Samoan mothers when they are asked to report their child’s physical activity preferences. Furthermore, mothers in Samoa on average have over twice as many children (total fertility rate (TFR): 4.3) [26] as mothers in high-income countries (TFR: 1.7) [27]. Larger families often mean less individualized attention for each child and may further reduce the accuracy of maternal reporting. We suspect that both shared childrearing and large families may have contributed specifically to the lack of discriminative ability observed for NPAQ Question 1 (prefers to play with other children/alone), as children are less likely to play alone in this setting than they may in others.

The lack of structured physical activity in this age group presents a unique challenge when attempting to estimate the physical activity of preschool-aged Samoan children. Question 3 in the NPAQ, which asks about the child’s attitude toward sports, may not be applicable to preschool-aged children who primarily engage in unstructured play with their peers rather than organized sports or structured exercise. This may be partly a result of the children’s early stage of development, but also because many sports programs require children to be older before they may participate. For example, the Samoa Sport for Development Program [28], an initiative that aims to improve health and village cohesion through participation in sports, is having success using sports to increase the physical activity of participants; however, the programs are only offered to children aged six years old and older. Additionally, the sport examples given in Question 3, soccer and basketball, are less relevant to Samoans in this age group than they may be in other settings; providing more culturally relevant examples such as rugby or cricket could potentially increase the usefulness of this question [29]. NPAQ Question 5, which asks if the child likes or dislikes reading, also may not be a culturally appropriate indicator of sedentary time in this age group. A study of 2–5-year-olds in Samoa found that in the three days prior to the interview, only 27.8% of caregivers had read or looked at books with their child [30], likely reflecting cost and general availability-related lack of access to children’s books in many Samoan homes.

In the case that an analysis is conducted with NPAQ data in this population, we recommend the use of the 15-point modified score over the full NPAQ score because of the more robust trends observed with accelerometer-measured physical activity levels. Our recommendation is also supported by the increase in Cronbach’s alpha from the full NPAQ score to the 15-point score despite the tendency of Cronbach’s alpha to decrease as the number of question items and the average interitem correlation decreases. For future investigations, qualitative interviews to better understand traits that Samoans associate with sedentary time and physical activity are essential for the development of an improved preference-based questionnaire.

This analysis had some limitations. Accelerometer-measured physical activity was only assessed in a small proportion of the total cohort, thus likely underpowering these analyses. This decision was driven purely by equipment constraints, although the characteristics of those who wore and did not wear the accelerometers were similar. Our findings may not be generalizable to the Samoan population as a whole due to the proportional recruitment of participants from each census region [13], which oversampled children from the Apia Urban Area in particular. Additionally, though accelerometer-measured physical activity did not differ between boys and girls in this population, child sex may have impacted mothers’ perception and reporting of physical activity; a larger sample size would allow us to stratify our analyses and explore this phenomenon. 

We caution against any comparison of the physical activity estimates reported here with other populations unless accelerometer devices were similarly wrist worn. While this is not a concern for this analysis, wrist-worn, rather than waist-worn, accelerometers tend to overestimate physical activity [31]. We acknowledge that if different intensities of cut points were used, our results for minutes of high-intensity activity and sedentary activity may have been different; CPM, however, would have been unaffected. Our choice of the Johansson cut points was based on the accelerometers being wrist-worn, whereas other cut points were derived from waist-worn accelerometry [32,33]. Physical activity measurements may additionally have been affected by the use of uniform sleep and wake times for the whole sample as opposed to individual diaries of sleep and wake times.

Despite these limitations, our study had several strengths. To our knowledge, this is the first time accelerometers have been used in the preschool age group in Samoa or the Pacific Island nations generally. These device-based measurements inform our understanding of preschool-aged children’s physical activity in the Samoan context. Extensive research has been conducted on the nutritional intake and diet of Samoan children [34]; however, the quantity and intensity of physical activity is poorly understood. With additional accelerometer data being collected in this ongoing cohort study, future investigations will focus on patterns of physical activity and their association with cardiometabolic health, as well as developing new, age-appropriate questionnaire tools for the measurement of physical activity in Samoan children as they grow up. Given the high levels of overweight/obesity observed among Samoan children [13] and evidence that physical activity habits track from early to late childhood [35], this will be important to address gaps in research and inform future interventions to reduce overweight and obesity in this population. 

## 5. Conclusions

Effectively measuring physical activity during child growth and development is important to inform preventative overweight/obesity interventions that emphasize healthy behaviors early in life and provide an evidence base for national physical activity guidelines. The poor concordance observed between accelerometer data and NPAQ scores indicate that neither the full NPAQ score nor our modified scores are useful measures of physical activity in children aged 2–4 years old in Samoa, although the 15-point modified score proved to be slightly more concordant with accelerometer data than both the full NPAQ scores and the 30-point modified score. Our findings underscore the value of accelerometer-measured physical activity, as well as the urgent need for the development of a culturally and age-appropriate physical activity questionnaire for use in this population. More broadly, this research raises several issues of relevance to planned and ongoing research in other settings—particularly under-resourced, economically disadvantaged and low-income international settings. Establishing culturally appropriate and accurate physical activity measurement approaches for use in these special populations is essential to establish setting-specific relationships between physical activity and public health. 

## Figures and Tables

**Table 1:** Child, mother and household characteristics by total sample and accelerometer subset.

Characteristics	Total (*n* = 319) N (%) or Mean ± SD	Accelerometer Subset (*n* = 61) N (%) or Mean ± SD	*p* ^1^
**Child**			
Age (years)	3.31 ± 0.89	3.45 ± 0.83	0.1673
Sex			0.3533
Male	165 (51.7)	27 (44.3)	
Female	154 (48.3)	34 (55.7)	
Attending School ^2^			1
Yes	55 (17.4)	11 (18.3)	
No	262 (82.6)	49 (81.7)	
**Mother**			
Age group (years)			0.1067
18–24.9	69 (21.6)	17 (27.9)	
25–39.9	163 (51.1)	27 (44.3)	
≥40	87 (27.3)	17 (27.9)	
Education (years)	12.30 ± 2.01	12.34 ± 1.50	0.8061
**Household**			
Asset score (range: 0–18)	5.64 ± 3.72	5.98 ± 3.83	0.4126
Census region			0.1841
Rest of Upolu	106 (33.2)	13 (21.3)	
Northwest Upolu	110 (34.5)	25 (41.0)	
Apia Urban Area	103 (32.3)	23 (37.7)	

^1^ *p*-values for *t*-test for continuous variables with normal distribution, Wilcoxon–Mann–Whitney test for continuous variables with non-normal distributions, Chi-Square test or Fisher’s Exact for categorical variables. ^2^ Counts do not sum to total. Two children missing from school attendance data.

**Table 2:** NPAQ response frequencies.

	1 N (%)	2 N (%)	3 N (%)	4 N (%)	5 N (%)	
Prefers to play alone	9 (2.8)	0 (0)	5 (1.6)	5 (1.6)	300 (94.0)	Prefers to play with others
Prefers vigorous games	216 (67.7)	21 (6.6)	47 (14.7)	10 (3.1)	25 (7.8)	Prefers quiet games
Dislikes playing sports	26 (8.2)	9 (2.8)	38 (11.9)	24 (7.5)	222 (69.6)	Likes playing sports
Is more introverted	17 (5.3)	6 (1.9)	45 (14.1)	23 (7.2)	228 (71.5)	Is more extroverted
Likes reading	55 (17.2)	36 (11.3)	162 (50.8)	38 (11.9)	28 (8.8)	Dislikes reading
Likes to play outside	179 (56.3)	18 (5.6)	52 (16.3)	14 (4.4)	55 (17.2)	Likes to play inside
Less physically active compared to other children	14 (4.4)	36 (11.3)	52 (16.3)	33 (10.3)	184 (57.7)	More physically active compared to other children

Note: Respondents were asked to score their child from 1 to 5 based on how strongly their child’s behavior aligns with either statement, with 1 typically corresponding with the less active option and 5 typically corresponding to the more active option, with the exception for Questions 2 and 6 that should be reverse scored.

**Table 3:** NPAQ question agreement: Spearman correlation coefficients and 95% confidence intervals.

Variable	*M*	*IQR*	Q1	Q2	Q3	Q4	Q5	Q6
Question 1	5	0						
Question 2 ^1^	5	2	0.30 * [0.16, 0.043]					
Question 3	5	1	0.16 * [0.04, 0.28]	0.09 [−0.02, 0.09]				
Question 4	5	1	0.25 * [0.11, 0.37]	0.34 * [0.22, 0.45]	−0.02 [−0.13, 0.08]			
Question 5	3	1	−0.03 [−0.14, 0.08]	−0.09 [−0.20, 0.03]	−0.02 [−0.13, 0.08]	−0.10 [−0.21, 0.00]		
Question 6 ^1^	5	2	0.15 * [0.03, 0.28]	0.31 * [0.20, 0.42]	0.09 [−0.03, 0.20]	0.26 * [0.15, 0.37]	0.14 * [0.02, 0.24]	
Question 7	5	2	0.08 [−0.04, 0.20]	0.00 [−0.10, 0.11]	0.25 * [0.14, 0.36]	−0.00 [−0.11, 0.11]	−0.07 [−0.18, 0.04]	0.10 [−0.02, 0.21]

^1^ Questions 2 and 6 reversed for correlation matrix. Note. *M* and *IQR* are used to represent median and interquartile range, respectively. Values in square brackets indicate the 95% confidence interval for each correlation. * indicates *p* < 0.05.

**Table 4:** Accelerometer-measured physical activity (EMM ± SE) according to individual NPAQ responses.

	N (%)	Average Daily CPM	High-Intensity Activity (min/day)	Sedentary Behavior (min/day)
Prefers to play with other children/alone				
1—alone	1 (1.6)	5513 ± 838	184 ± 36.23	266 ± 55.10
3	1 (1.6)	5444 ± 841	169 ± 36.38	269 ± 55.33
5—with others	59 (96.7)	4359 ± 108	124 ± 4.69	351 ± 7.13
Prefers vigorous/quiet games				
1—quiet games	2 (3.3)	4519 ± 588	130 ± 25.5	319 ± 39.48
2	1 (1.6)	5539 ± 831	177 ± 36.0	285 ± 55.81
3	13 (21.3)	4000 ± 233	108 ± 10.1	369 ± 15.62
4	2 (3.3)	4142 ± 588	114 ± 25.5	362 ± 39.49
5—vigorous games	43 (70.5)	4495 ± 127	130 ± 5.5	344 ± 8.52
Likes/dislikes playing sports				
1—dislikes sports	7 (11.5)	4976 ± 304	156 ± 12.94	325 ± 20.61
2	1 (1.6)	3627 ± 803	97 ± 34.20	393 ± 54.49
3	5 (8.2)	4452 ± 357	123 ± 15.19	341 ± 24.20
4	5 (8.2)	5148 ± 358	160 ± 15.26	298 ± 24.31
5—likes sports	43 (70.5)	4225 ± 122	117 ± 5.19	358 ± 8.27
Is more extroverted/introverted				
1—introverted	2 (3.3)	4324 ± 580	127 ± 25.46	354 ± 39.16
2	1 (1.6)	4610 ± 809	121 ± 35.52	304 ± 54.64
3	10 (16.4)	3705 ± 256	97 ± 11.24	387 ± 17.29
4	2 (3.3)	4725 ± 591	131 ± 25.94	327 ± 39.90
5—extroverted	46 (75.4)	4530 ± 119	131 ± 5.24	342 ± 8.05
Likes/dislikes reading				
1—likes reading	12 (19.7)	4410 ± 244	130 ± 10.71	355 ± 16.2
2	9 (14.8)	4796 ± 283	139 ± 12.38	321 ± 18.7
3	26 (42.6)	4226 ± 157	119 ± 6.88	357 ± 10.4
4	6 (9.8)	4409 ± 346	121 ± 15.17	344 ± 23.0
5—dislikes reading	5 (8.2)	4607 ± 381	133 ± 16.71	337 ± 25.3
Likes to play outside/inside				
1—inside play	9 (14.8)	4252 ± 283	119 ± 12.30	357 ± 18.89
2	3 (4.9)	4298 ± 491	116 ± 21.33	343 ± 32.76
3	10 (16.4)	4129 ± 268	114 ± 11.67	364 ± 17.92
4	3 (4.9)	4997 ± 494	151 ± 21.46	314 ± 32.97
5—outside play	36 (59.0)	4464 ± 142	129 ± 6.16	345 ± 9.46
More/less physically active than others				
1—less active	2 (3.3)	5502 ± 587	162 ± 25.52	260 ± 38.69
2	7 (11.5)	4125 ± 312	109 ± 13.55	360 ± 20.54
3	10 (16.4)	4717 ± 260	144 ± 11.33	335 ± 17.17
4	6 (9.8)	4501 ± 338	129 ± 14.68	337 ± 22.26
5—more active	36 (59.0)	4280 ± 137	121 ± 5.97	357 ± 9.05

CPM: counts per minute, EMM: estimated marginal means, SE: standard error.

**Table 5:** Accelerometer-measured physical activity (EMM ± SE) by modified NPAQ scores.

	N (%)	Average Daily CPM	High-Intensity Activity (min/day)	Sedentary Behavior (min/day)
**15 pt NPAQ** ^1^				
Likert grouped tertiles				
1–6	2 (3.3)	4352 ± 611	128 ± 26.58	353 ± 40.62
7–12	17 (27.9)	4284 ± 207	120 ± 9.02	352 ± 13.79
13–15	42 (68.9)	4063 ± 132	127 ± 5.73	347 ± 8.75
Evenly distributed tertiles				
1–12	19 (31.1)	4291± 195	121 ± 8.47	352 ± 13.00
13–14	16 (26.2)	4334 ± 214	121 ± 9.27	353 ± 14.20
15	26 (42.6)	4510 ± 167	131 ± 7.27	343 ± 11.10
Likert grouped quartiles				
1–8	7 (11.5)	3813 ± 316	97.9 ± 13.65	375 ± 21.3
9–11	11 (18.0)	4481 ± 252	130.7 ± 10.89	343 ± 17.0
12–14	17 (27.9)	4409 ± 204	124.7 ± 8.79	349 ± 13.7
15	26 (42.6)	4507 ± 164	130.9 ± 7.10	343 ± 11.1
**30 pt NPAQ** ^1^				
Likert grouped tertiles				
1–12	1 (1.6)	5489 ± 849	183 ± 36.70	268 ± 56.02
13–24	32 (52.5)	4337 ± 149	124 ± 6.46	353 ± 9.86
25–30	28 (45.9)	4423 ± 160	125 ± 6.92	346 ± 10.57
Evenly distributed tertiles				
1–21	18 (29.5)	4353 ± 202	122 ± 8.78	349 ± 13.4
22–25	18 (29.5)	4397 ± 202	128 ± 8.77	350 ± 13.4
26–30	25 (41.0)	4426 ± 172	126 ± 7.46	346 ± 11.4
Likert grouped quartiles				
1–15	1 (1.6)	5500 ± 852	183 ± 36.68	268 ± 56.5
16–20	12 (19.7)	4194 ± 245	115 ± 10.54	356 ± 16.2
21–25	23 (37.7)	4424 ± 178	128 ± 7.65	350 ± 11.8
26–30	25 (41.0)	4422 ± 170	126 ± 7.31	346 ± 11.3
Evenly distributed quartiles				
1–20	13 (21.3)	4296 ± 238	120 ± 10.34	349 ± 15.8
21–23	12 (19.7)	4462 ± 249	131 ± 10.82	352 ± 16.5
24–26	21 (34.4)	4317 ± 187	122 ± 8.13	353 ± 12.4
27–30	15 (24.6)	4539 ± 222	130 ± 9.62	338 ± 14.7

^1^ The 15-point score includes Questions 2, 4 and 6. The 30-point score includes Question 1 through Question 6. CPM: counts per minute, EMM: estimated marginal means, SE: standard error.

## Data Availability

Data are available upon reasonable request to the corresponding author.

## Appendix A. Netherlands Physical Activity Questionnaire and Accompanying Samoan Translation

**Mo fesili o i lalo ifo, fa’amolemole li’o le numera e fa’amatala ai [igoa] i le ono masina ua tuana’i atu nei. Fa’ata’ita’iga, afai e fiafia [igoa] e taalo to’atasi nai lo’o le taalo faatasi ma isi tamaiti, li’o le numera 3 mo le fesili muamua. Afai e fiafia lou alo e taalo ma isi tamaiti nai lo’o le taalo na o ia, li’o le numera 5.**

For the following questions, please circle the number that best describes [name] during the past six months. For example, if [name] preferred to play alone as often as he/she preferred to play with other children, circle the number 3 for the first question. On the other hand, if he or she almost always preferred playing with other children, rather than alone, circle the number 5.

Almost always	About Equal					Almost always
**Tele o taimi**	**Isi taimi**					**Tele o taimi**
**E fiafia e taalo na o ia**	1	2	3	4	5	**E fiafia e taalo ma isi tamaiti**
Prefers to play alone						Prefers to play with other children
**E fiafia taaloga e malosi**	1	2	3	4	5	**E fiafia taaloga filemu**
(e.g., tag, kickball)						(e.g., board games)
Prefers vigorous games						Prefers quiet games
**E lē fiafia e taalo i ta’aloga**	1	2	3	4	5	**E fiafia e taalo i ta’aloga**
(e.g., soccer, basketball)						Likes playing sports
Dislikes playing sports						
**E filemu**	1	2	3	4	5	**E tautalatala**
(e.g., quiet, reserved)						(e.g., outgoing)
Is more introverted						Is more extroverted
**E fiafia e faitau**	1	2	3	4	5	**E lē fiafia e faitau**
Likes to read						Dislikes reading
**E fiafia e taalo i fafo**	1	2	3	4	5	**E fiafia e taalo i totonu o le fale**
Likes to play outside						Likes to play inside (home/school)
**E lē fiafia a gaioi e pei o isi**	1	2	3	4	5	**E fiafia e gaioi e pei o isi**
**tamaiti e tutusa o latou tausaga**						**tamaiti e tutusa o latou tausaga**
Less physically active compared						More physically active compared
to children of the same age						to children of the same age
